# The General Practitioner—His Book

**Published:** 1911-03-04

**Authors:** 


### The General Practitioner—His Book.

Few books in medicine can be read with interest by all classes of the profession. Those few which can, are books for all time and not for any fixed period. The works of Hippocrates, of Sir Thomas Browne, of Wiseman, Guy de Chauliac, John Hilton, Bilroth, Laennec—to mention a few only—are directly read by few men nowadays, but they are indirectly read by everyone through the works of others. This is because they contain broad principles of practice and of precept which all acknowledge and try to act up to without distinctly knowing the works in which they originally appeared. But the majority of books can appeal only to one class of medical men, and those appealing to the general practitioner will necessarily be the most widely distributed and read.

What, then, are the essentials for any book which is written to interest and to help the general practitioner? It is easy enough to enumerate a few indispensable characteristics. For instance, the book must be short and concise. The general practitioner is essentially a busy man. We do not mean by this that he works much, or any, harder than does the consultant; but to the latter reading is a part of his daily work, for he must not only keep up with the time, but he must try to get ahead of it; to the former on the other hand, reading is a part of his relaxation, he can only read when all his patients have been seen, when all his books have been written, and when all his medicines have been made up and sent off; even then he can only do so with one ear on the telephone and with an ever alert apprehension of the night-bell. Then, again, the common cases to the specialist are the rare cases to the general practitioner, and have the same interest to the latter that the pathological curiosities have to the former. The general practitioner must be somewhat of a specialist in all branches of medicine, and he wants to know which are the minor cases in each branch that he can treat, and which are the major ones that he must send on to the specialist. His text book therefore should divide the diseases with which it treats into those that he can manage by himself, and those that he can only treat after consultation with, and under the advice of, a man more experienced in the special branch. The first type cannot be described in too much detail of diagnosis, of pathology, and of treatment; the second need only be indicated, so that he may not treat a major malady as a minor and so allow the time to go by in which successful interference is possible before the case has passed into an irremediable condition. Again, the cases should be approached from a clinical or symptomatic rather than from a pathological standpoint. The practitioner sees before him a case of difficulty in swallowing or of discharge from the nose, and wants to have in his mind the common and rare causes of these symptoms rather than to have at his fingers' end the pathological ramifications of a case of growth of the œsophagus or of sinusitis. For this reason the subject is often best approached from the class of patient before him than from the organ in which the disease arises; in the child there is no need to think of growth of the œsophagus when considering dysphagia, nor in an old patient to think of adenoids as a primary cause of discharge from the nose. If an author bear these points in view he will necessarily be dogmatic; this may bring criticism upon him by scientific purists. He will lay a theory before his readers as a scientific fact when he knows that there are criticisms that may be made against it. He must do so, however, in order to give a working hypothesis on which to base the treatment; and as the treatment would not be altered by the criticisms, it is unnecessary to mask the clearness of the account and to spoil its beauty by introducing them. He may use the word "always" occasionally while he knows there are rare exceptions to the rule, since the exceptions may be so few that they do not affect the practical application.

Judged by criteria such as these, we have before us a book which should be of great use to the general practitioner. It is called "Hints for the General Practitioner in Rhinology and Laryngology," by Dr. Johann Feiss, of Vienna. By means of a complete contents table and index the reader can readily refer to any subject. He will find the simple and the complex maladies carefully kept apart, and a useful chapter on examination. The book has been translated into readable English by Mr. Bowring Horgan.

